# Net age, but not integrity, may be associated with decreased protection against *Plasmodium falciparum* infection in southern Malawi

**DOI:** 10.1186/s12936-019-2930-8

**Published:** 2019-09-24

**Authors:** Liana R. Andronescu, Andrea G. Buchwald, Jenna E. Coalson, Lauren Cohee, Andy Bauleni, Jenny A. Walldorf, Chifundo Kandangwe, Themba Mzilahowa, Terrie E. Taylor, Don P. Mathanga, Miriam K. Laufer

**Affiliations:** 1 Center for Vaccine Development and Global Health, University of Maryland School of Medicine, 685 W, Baltimore Street, Baltimore, MD 21201 USA; 2 Mel and Enid Zuckerman College of Public Health, University of Arizona, 1295N Martin Ave, Tucson, AZ 85724 USA; 3 Malaria Alert Centre, University of Malawi College of Medicine, Blantyre, Malawi; 4 Department of Osteopathic Medical Specialties, Michigan State University, 909 Fee Road, East Lansing, MI 48824 USA; 5 Present Address: University of Colorado School of Public Health, University of Colorado, 13001 E, 17th Place, Mail Stop B119, Aurora, CO 80045 USA; 6 Present Address: Global Immunization Division, Center for Global Health, Centers for Disease Control and Prevention, Atlanta, GA USA

**Keywords:** ITN, Malawi, Efficacy, Malaria control

## Abstract

**Background:**

Distribution campaigns for insecticide-treated nets (ITN) have increased the use of ITNs in Malawi, but malaria prevalence remains high even among those using the nets. Previous studies have addressed ITN ownership, insecticide resistance, and frequency of ITN use as possible contributing factors to the high prevalence of malaria infection despite high ITN coverage, but have rarely considered whether the condition of the ITN, or how many people use it, impacts efficacy. This study assessed how ITN integrity, ITN age, and the number of persons sharing a net might mitigate or reduce protective efficacy among self-identified ITN users in Malawi.

**Methods:**

From 2012 to 2014, six cross-sectional surveys were conducted in both the rainy and dry seasons in southern Malawi. Data were collected on ITN use, integrity (number and size of holes), and age. Blood samples for detecting *Plasmodium falciparum* infection were obtained from reported ITN users over 6 months of age. Generalized linear mixed models were used to account for clustering at the household and community level. The final model controlled for gender, household eaves, and community-level infection prevalence during the rainy season.

**Results:**

There were 9646 ITN users with blood samples across six surveys, 15% of whom tested positive for *P. falciparum* infection. Among children under 5 years old, there was a 50% increased odds of *P. falciparum* infection among those sleeping under an ITN older than two years, compared to those using an ITN less than 2 years old (OR = 1.50; 95% CI 1.07–2.08). ITN integrity and number of individuals sharing an ITN were not associated with *P. falciparum* infection.

**Conclusions:**

Older ITNs were associated with higher rates of *P. falciparum* in young children, which may indicate that insecticide concentrations play a larger role in infection prevention than the physical barrier of an ITN. ITN use was self-reported and the integrity measures lacked the precision of newer methods, suggesting a need for objective measures of ITN use and more precise assessment of ITN integrity.

## Background

The World Health Organization reported 219 million malaria cases in 2017, resulting in 435,000 deaths worldwide [1]. Malaria remains a leading cause of morbidity and mortality among children under five [1]. Insecticide-treated nets (ITNs) are a cornerstone of malaria control programs across the region and have decreased disease due to *Plasmodium falciparum* infection in countries across sub-Saharan Africa [2–4]. The ITN provides a physical barrier against mosquitoes and contains an insecticide which can both repel and kill mosquitoes before they reach people beneath the net [5]. Ideally, ITNs prevent mosquitoes from transmitting the parasite and, with sufficient coverage, data suggest that overall malaria prevalence can decrease [6–8]. There are several examples throughout sub-Saharan Africa that have not shown the expected decrease of malaria prevalence in association with increased use of ITNs [9–11].

Malawi is an example of a country where this phenomenon has been observed. In 2012, an estimated 5.6 million ITNs were distributed to households across Malawi, achieving the targeted goal of one ITN per every two people in 58% of households. [12–14]. This distribution provided more national coverage than had previously been achieved. Despite the distribution campaign improving access to and use of ITNs, the burden of *P. falciparum* infections did not lead to any further recent decreases in malaria in Malawi [15]. The Malaria Indicator Survey from 2012 reported a nationwide malaria prevalence of 28%, but by the 2014 report the prevalence had increased to 33% [12, 16]. Furthermore, in a recent study in southern Malawi, it was demonstrated that ITN use was associated with only modest protection against *P. falciparum* infections among ITN users, which is consistent with the steady *P. falciparum* prevalence rates reported after the ITN distribution campaign [17].

The reason for the limited impact of ITN access and reported use on malaria prevalence remains unknown. It was hypothesized that previously unmeasured features of the ITNs may be reducing their protective efficacy. As ITNs age, efficacy may wane because (a) the concentration of insecticide decreases and limits the repellent/killing effect, or (b) the net develops holes large enough for mosquitoes to pass through, diminishing the physical barrier [18]. Since the reported use of ITNs was not shown to consistently protect against malaria infection, the study was designed to test the hypothesis that ITN characteristics such as quality or age, or high numbers of people using a single ITN may explain their relative failure to reduce *P. falciparum* infections among ITN users [19, 20]. Utilizing cross-sectional data collected over 3 years in southern Malawi, there was a robust data set to test the hypothesis and construct a model to evaluate why ITNs are not performing as expected.

## Methods

### Survey

Data came from six cross-sectional household surveys conducted in southern Malawi, which were conducted to assess malaria prevalence. Details of the survey methods have been previously reported [17]. Two surveys, the first at the end of the rainy season (high transmission months of April–May), and the second at the end of the dry season (low transmission months of September–October), were conducted each year from 2012 to 2014, as described previously [21, 22]. Approximately 300 households were selected from each of three districts in southern Malawi: Blantyre (an urban, low transmission setting), Chikhwawa (a rural, low altitude, high transmission setting), and Thyolo (a rural, high altitude, low transmission setting) using two-stage cluster sampling. There were 10 enumeration areas (EAs) randomly selected from each district and households were selected from each EA by compact segment sampling. Each selected EA was divided into communities containing approximately 30 households. One cluster in the compact segment from each EA was randomly selected and all households within that cluster were visited. The same selected compact segments in each district were surveyed each season, and all households within a given compact segment were visited on a single day. Households were excluded if there were no adults over 18 years old present to provide consent. If excluded, a household was replaced with the nearest household within the compact segment, selected by convenience, so that the desired number of households were sampled. Households in the same geographic area were selected in each survey, and the same households may have been visited repeatedly, however, identifying data to track individuals or households between surveys was not collected.

### Ethical treatment of human subjects

Prior to study initiation, permission to survey each village was provided by the village leaders. Written informed consent was obtained from all adults (18 years or older) and guardians of children and assent was obtained from children age 13–17 years. All questionnaires were administered in the local language, Chichewa. The study received ethical approval from the University of Maryland School of Medicine and Michigan State University Institutional Review Boards and the University of Malawi College of Medicine Research and Ethics Committee.

### Data collection

Survey questionnaires were adapted from the standardized Malaria Indicator Survey tools [23]. Data were collected on Android-based tablets using OpenDataKit and managed using Research Electronic Data Capture (REDCap) tools [24]. Data collected included household characteristics and socio-economic indicators, individual demographics, household net ownership and individual net use. Participant age and gender were collected. Participants were initially classified into three age groups, defined a priori, for preliminary analysis: young children (6 months to 5 years), school-aged children (children 5 to 15 years old), and adults (over 15 years). Participants were included for analysis if they were identified as using an ITN the night before the survey and had a blood specimen result available.

Community prevalence of infection was calculated as an average of the infection prevalence across all rainy season surveys, to control for geographic differences in parasite transmission rates. Open or closed eaves on a residence, which can increase mosquito exposure, were reported based on observations by the interviewer.

ITN use was based on the head of household reporting use of an ITN during the night prior to the interview and identifying which household member slept under which net. The age of each ITN in years was also self-reported and categorized into less than 1 year, 1 to 2 years, and more than 2 years. This was subsequently collapsed into a dichotomous variable when analysis showed no statistically significant difference in prevalence between ITNs less than a year old or between 1 and 2 years old. ITN integrity was established by a fieldworker who quantified the size and number of holes. The quality was categorized as good (no holes), fair (no holes larger than a size D battery with a 33 mm diameter), poor (1–4 holes that fit a size D battery with a 33 mm diameter), or very poor (5 + holes that fit a size D battery with a 33 mm diameter) [25]. The number of individuals who share an ITN was based how many household members the head of household reported using the same ITN the previous night.

### Malaria diagnosis

Blood specimens were collected from household members over 6 months of age who were present at the time of survey. Study nurses collected thick blood smears on slides for microscopy and dried blood spots on 3 M Whatman filter papers for quantitative PCR (qPCR). Participants were considered to have *P. falciparum* infection if the qPCR was positive for *P. falciparum* parasitaemia lactate dehydrogenase gene (limit of detection, 0.5–5 parasites/μL) or blood smear results determined presence of microscopically detectable infection using thick blood smears stained with Field’s or Giemsa, that was confirmed by at least two readers [17, 26].

### Statistical analysis

Univariate and bivariate analyses were conducted to describe the study sample. Multivariable mixed effect logistic regression (SAS PROC GLIMMIX) was used to model the association between *P. falciparum* infection and ITN age and integrity. ITN age and integrity were assessed as individual exposures and tested together for potential interaction. The analyses accounted for clustering at the household and community level using nested random intercepts. Covariates investigated for a relationship with *P. falciparum* infection status included participant age, gender, number of individuals sharing an ITN, open or closed eaves on the home, and community-level *P. falciparum* prevalence. Interaction terms for modification of the association between exposures of interest and *P. falciparum* infection by age and by community-level rainy season prevalence were also assessed because of prior data showing different infection risk by age [17]. All analyses were conducted using SAS 9.4.

## Results

Among the 26,018 participants enrolled in the six surveys, 12,075 were classified as ITN users and 9646 also had blood specimen results available (Table 1). Only participants with net use and infection status data were included for this secondary analysis. Less than half (37%) of ITN users with test results were males and 22% were children under 5 years old. Among users with test results, 3088 ITNs (32%) were more than 2 years old, and 2824 (29%) of ITNs were rated as having poor or very poor integrity. Participants who reported sleeping with only one other person under an ITN made up 45% (n = 4293) of ITN users and 1565 (16%) reported sharing an ITN with four or more household members. Open eaves were reported in the households of 2379 (25%) ITN users. All covariates measured were significantly different (p < 0.05) between the population who used ITNs and had test results and those who used ITNs but did not have test results. ITN users without test results were more likely to be male (73%) and over the age of 15 (65%). There were some records missing ages, which is not uncommon for a large cross-sectional survey in settings where birthdates may not be known. Records missing gender data were otherwise complete and the missingness is likely due to data entry error.

**Table 1:** Descriptive characteristics of ITN users in six cross-sectional surveys, Malawi 2012–2014

Characteristic	ITN users with test results (N = 9646) n (%)
*P. falciparum* (PCR result)	
Positive	1460 (15)
Negative	8186 (85)
*Missing*	0 (0)
Sex	
Male	3582 (37)
Female	6041 (63)
*Missing*	23 (0)
Age	
0–5 years	2128 (22)
5–15 years	2777 (29)
> 15 years	4709 (49)
*Unknown*	32 (0)
ITN age	
0–2 years	6558 (68)
2+ years	3088 (32)
ITN integrity	
Good	4300 (45)
Fair	2522 (26)
Poor	1723 (18)
Very poor	1101 (11)
Persons sharing an ITN	
1 or 2	4293 (45)
3	3788 (39)
4+	1565 (16)
Eaves	
Open	2379 (25)
Closed	7264 (75)
*Missing*	3 (0)
Community infection prevalence	
< 6%	1888 (20)
6–9%	4632 (48)
> 9%	3126 (32)

### Unadjusted analysis

The impact of bed net integrity on protection against infection was evaluated by comparing the prevalence of *P. falciparum* infection among participants with ITNs in each of the four categories: good, fair, poor, and very poor. There were no significant differences between the good and fair groups and the poor and very poor groups, so those categories were combined for further analysis. Among participants who slept under an ITN of poor or very poor integrity, 428 (15%) tested positive for *P. falciparum* infection and 1032 (15%) of those sleeping under a good or fair quality ITN tested positive, with an unadjusted odds ratio of 1.00 (95% CI 0.89–1.13) (Table 2). Although this measure of ITN integrity was not associated with *P. falciparum* infection, it was included in the final model.

**Table 2:** Unadjusted associations between potential predictors, covariates, and *P. falciparum* infection using a generalized linear mixed model

	Number with *Plasmodium* infection (%)	Crude OR (95% CI)
ITN integrity		
Good/fair	1032 (15)	REF^a^
Poor/bad	428 (15)	1.00 (0.89, 1.13)
ITN age		
0–2 years	1009 (15)	REF
2+ years	451 (15)	0.94 (0.83, 1.06)
Persons sharing an ITN		
1 or 2	559 (13)	REF
3	609 (16)	1.14 (0.94, 1.38)
4+	292 (19)	1.36 (1.10, 1.69)
Sex		
Male	597 (17)	REF
Female	862 (14)	0.85 (0.77, 0.94)
Age		
0–5 years	252 (12)	REF
5–15 years	621 (22)	2.14 (1.83, 2.51)
> 15 years	581 (12)	1.05 (0.90, 1.23)
Eaves		
Open	538 (23)	REF
Closed	922 (13)	0.42 (0.35, 0.50)
Community infection prevalence		
< 6%	64 (3)	REF
6–9%	398 (9)	2.68 (2.05, 3.51)
> 9%	998 (32)	13.37 (10.30, 17.34)

^a^REF is the reference odds ratio of 1.00

Age of the ITN was also not associated with its protective efficacy. Users of older ITNs (≥ 2 years) had the same prevalence of *P. falciparum* infection as users of newer ITNs (< 2 years) (OR = 0.94; 95% CI 0.83–1.06, Table 2).

Participants that reported four or more household members sharing a single ITN had a *P. falciparum* prevalence of 19%, corresponding to 36% higher odds *P. falciparum* infection compared to those who slept with one other person under an ITN (OR = 1.36; 95% CI 1.10–1.69, Table 2). Number of persons sleeping under the net was also included in the final model.

### Final model

The final model includes number of persons sharing the ITN, eaves, participant gender and rainy season infection prevalence. The associations between ITN age and integrity showed no evidence of interaction and were combined into a single model. The model was stratified by age due to evidence of interaction between participant age and ITN age on *P. falciparum* infection. School aged children and adults were combined into a single group for the final model because their results were not statistically different from each other and ITN age was recategorized into a binary variable (Table 3).

**Table 3:** Final model of predictors of *P. falciparum* infection, stratified by participant age using a generalized linear mixed model

	6 months–5 years	5+ years
	aOR (95% CI)^a^	aOR (95% CI)
ITN integrity		
Good/fair	REF	REF
Poor/bad	1.10 (0.78, 1.55)	1.06 (0.90, 1.25)
ITN age		
< 2 years	REF	REF
2+ years	1.50 (1.07, 2.08)	0.80 (0.68, 0.92)
Number sharing the net		
1 or 2	REF	REF
3	1.25 (0.83, 1.89)	1.22 (1.06, 1.41)
4+	1.25 (0.80, 1.95)	1.10 (0.91, 1.33)

Adjusted for sex, community infection prevalence, and eaves. Model adjusted for clustering at the community level

^a^Adjusted odds ratio

After adjusting for gender, eaves, and community infection prevalence, there was 50% increased odds of *P. falciparum* infection among children under 5 years old sleeping under an older ITN (> 2 years), compared to children under 5 years old using a newer ITN (OR = 1.50, 95% CI 1.07, 2.08). Among children over 5 years old and adults there were 20% reduced odds of *P. falciparum* infection for those using an older ITN compared to those using a newer ITN (95% CI 0.68, 0.92). There was no statistically significant association between the number of people sharing an ITN and the odds of *P. falciparum* infection.

## Discussion

This study found that the age of an ITN may play a larger role in malaria prevention than ITN integrity, since there was no association between ITN integrity and *P. falciparum* infection. The results indicate that after 2 years, ITNs decrease in effectiveness among children under five. However, this association was reversed in participants over 5 years old, with older nets appearing to have a protective quality. These results suggest the presence of unmeasured confounding variables such as when the ITN is used, the concentration of insecticide in the ITN, or the location of holes on the ITN, none of which were addressed in the questionnaires used in these studies. Furthermore, the possibility that the results were found by chance cannot be excluded, though it is unlikely given the large sample size, and highlights the need for further investigation. The generalizability of these results to the whole population is impacted by the absence of blood test results for many male adults. The results of this study, with a robust sample size, are consistent with recent findings from a smaller study in a different location in Malawi that showed no association between physical integrity of ITNs and malaria [4].

The observed association between ITNs older than 2 years and *P. falciparum* infection in children under 5 years is also consistent with the current literature. Previous studies indicate that the efficacy of an ITN is close to 2 years. Current distribution campaigns are scheduled with the expectation that ITNs last 3 to 5 years, however more frequent replacement of ITNs is recommended [27–33]. The ITN distribution campaigns in Malawi are currently conducted every 3 years, which may be insufficient to maintain decreases in infection prevalence in children under 5 years. This study found that the age of an ITN seems more important for prevention of infection than the ITN integrity, which is similar to prior studies that indicate insecticide used on the ITNs is more important than the physical barrier in prevention of malaria [20, 34, 35]. Although these variables were not assessed in the surveys, different ITN brands and varying maintenance practices, such as washing and repairing of ITNs, may contribute to waning protective efficacy over time [36–40]. These are important issues to address in regions where ITNs are used regularly but insecticide resistance is increasing [19, 41].

The results of this analysis suggest that the failure of ITNs to protect against *P. falciparum* infection may lie in factors other than the age and integrity of the nets, as measured during this study. One explanation for low ITN efficacy may be that biting occurs at times when individuals are not under the bed net, but rather earlier in the evening and later in the morning than previously described. Previous studies have demonstrated that even when ITNs are used, individuals may still be exposed to mosquito bites in the early evening before the nets are used [42, 43]. Nets may also be less effective if not tucked in properly or lifted multiple times over the course the night [10]. ITNs will also be less effective if mosquitoes in the area are resistant to the pyrethroid used in the net [41]. Finally, behavioural factors must be considered to fully understand the effect of net age and quality. Studies to determine if ITNs are being used optimally may shed light on why ITN distribution campaigns alone do not provide protection.

It is also likely that ITN age and integrity are important measures of ITN efficacy but due to study methodology were not well captured. The primary limitation of this study is that the definition of ITN use relied on self-report. ITN use is difficult to measure accurately since participants may over-report desired behaviours and observing the presence of a hanging net does not confirm its nightly use. The self-report approach to measuring ITN adherence overestimates the actual rate of use, a common logistical limitation of surveillance efforts [44]. One more accurate measurement uses observations from unannounced night visits; however, this is difficult to implement and often unpopular because of privacy concerns. Another method of measuring use involves new ITN products that sense when the net is folded or unfurled [45]. Whichever data collection method is employed, multiple observations should be taken to establish a pattern of use, since ITN use can vary nightly or even seasonally [46]. This limitation points to the need for additional research into new methods for quantifying ITN use.

ITN integrity was assessed by counting holes of a specified size, as used in the Malaria Indicator Surveys, instead of employing more precise techniques such as the Proportional Hole Index (PHI), which is recommended by the World Health Organization and common in studies of net integrity [47]. The PHI has multiple measures of hole size and accounts for both the number of holes and the respective sizes of each hole, thus capturing the approximate total surface area of the ITN that is compromised. Other options involve assessing digital photos of each side of a net or using a ruler to measure the length, width, and location of each hole on a subset of ITNs [18]. It is also important to note the orientation of the holes on the ITN because mosquitoes are more likely to enter through the top than from the side, making holes on the roof of the ITN potentially more problematic than holes on the walls [4, 48]. By only measuring the number of holes of a certain size, the studies used for this analysis may not have quantified ITN integrity sufficiently to assess the true impact on protection. Overall, more detailed measurement of the size and location of holes would allow for more precision in the analysis of the association between ITN integrity and *P. falciparum* infection.

## Conclusion

This study found that older ITNs were associated with higher rates of *P. falciparum* among children under five, which may indicate that insecticide concentrations play a larger role in infection prevention than the physical barrier of an ITN. The significant role of insecticides in ITNs should be taken under consideration for designing policies in regions where insecticide resistance is spreading. New ITNs should be distributed with enough frequency to ensure families are less likely to rely on old nets and alternative insecticides should be assessed for potential use in ITNs to maximize insecticide efficacy. Future studies enrolling ITN users should include more precise measures of ITN integrity, and objective definitions of ITN use, as the claim of using an ITN the night prior to the interview may not be sufficient to characterize use.

## Data Availability

The datasets used and/or analysed during the current study are available from the corresponding author upon appropriate request and approval from the University of Malawi College of Medicine Research and Ethics Committee.

## Abbreviations

ITN: insecticide-treated net
EA: enumeration area
qPCR: quantitative polymerase chain reaction
CI: confidence interval
OR: odds ratio
